# A Hybrid Convolutional–Transformer Approach for Accurate Electroencephalography (EEG)-Based Parkinson’s Disease Detection

**DOI:** 10.3390/bioengineering12060583

**Published:** 2025-05-28

**Authors:** Chayut Bunterngchit, Laith H. Baniata, Hayder Albayati, Mohammad H. Baniata, Khalid Alharbi, Fanar Hamad Alshammari, Sangwoo Kang

**Affiliations:** 1 Division of Industrial and Logistics Engineering Technology, Faculty of Engineering and Technology, King Mongkut’s University of Technology North Bangkok, Rayong Campus, Rayong 21120, Thailand; chayut.b@eat.kmutnb.ac.th; 2 School of Computing, Gachon University, Seongnam 13120, Republic of Korea; 3 Endicott College, Woosong University, Daejeon 34606, Republic of Korea; hayder1111@wsu.ac.kr; 4 Department of Computer Science, Faculty of Information Technology, The World Islamic Sciences and Education University, Amman 11947, Jordan; mohammad.baniata@wise.edu.jo; 5 Department of Computer Science, College of Science, Northern Border University, Arar 91431, Saudi Arabia; khalid.nawaf@nbu.edu.sa; 6 Department of Biological Sciences, College of Science, Northern Border University, Arar 91431, Saudi Arabia; fanar.alshammari@nbu.edu.sa

**Keywords:** Parkinson’s disease, EEG, convolutional neural networks, transformer model, long short-term memory, deep learning

## Abstract

Parkinson’s disease (PD) is a progressive neurodegenerative disorder characterized by motor and cognitive impairments. Early detection is critical for effective intervention, but current diagnostic methods often lack accuracy and generalizability. Electroencephalography (EEG) offers a noninvasive means to monitor neural activity, revealing abnormal brain oscillations linked to PD pathology. However, deep learning models for EEG analysis frequently struggle to balance high accuracy with robust generalization across diverse patient populations. To overcome these challenges, this study proposes a convolutional transformer enhanced sequential model (CTESM), which integrates convolutional neural networks, transformer attention blocks, and long short-term memory layers to capture spatial, temporal, and sequential EEG features. Enhanced by biologically informed feature extraction techniques, including spectral power analysis, frequency band ratios, wavelet transforms, and statistical measures, the model was trained and evaluated on a publicly available EEG dataset comprising 31 participants (15 with PD and 16 healthy controls), recorded using 40 channels at a 500 Hz sampling rate. The CTESM achieved an exceptional classification accuracy of 99.7% and demonstrated strong generalization on independent test datasets. Rigorous evaluation across distinct training, validation, and testing phases confirmed the model’s robustness, stability, and predictive precision. These results highlight the CTESM’s potential for clinical deployment in early PD diagnosis, enabling timely therapeutic interventions and improved patient outcomes.

## 1. Introduction

A progressive neurodegenerative disorder, Parkinson’s disease (PD) profoundly impairs motor and non-motor functions, substantially reducing quality of life [1]. The World Health Organization estimates 8.5 million new PD diagnoses annually, a number projected to grow with increasing life expectancies [2,3]. Beyond its impact on individuals, PD imposes a notable socioeconomic burden, necessitating long-term care and straining healthcare systems [4]. Clinically, PD manifests through hallmark symptoms such as tremors, rigidity, bradykinesia, and postural instability [5], which diminish independence, elevate medical costs, and increase reliance on caregivers. Thus, early diagnosis is critical to mitigate both individual suffering and societal costs.

PD arises from the degeneration of dopaminergic neurons in the substantia nigra, disrupting basal ganglia pathways essential for motor control. This dopamine depletion triggers neural signaling imbalances, manifesting as hallmark motor symptoms. Beyond motor deficits, PD is linked to cognitive and neuropsychiatric impairments, including executive dysfunction, memory deficits, apathy, depression, and anxiety [6]. Emerging research also highlights PD’s impact on serotonin and norepinephrine systems, contributing to non-motor symptoms such as sleep disturbances and autonomic dysfunction. At the neural level, altered oscillatory activity, particularly in the beta frequency band (13–30 Hz), reflects disruptions in cortico-basal ganglia circuits, providing detectable biomarkers for tracking PD progression [7].

Electroencephalography (EEG) offers a powerful noninvasive method for detecting neurophysiological changes in PD. By recording oscillatory brain activity, EEG identifies biomarkers of neural dysregulation linked to cognitive and motor impairments [8,9]. Standard electrode systems facilitate the analysis of frontal and central brain regions commonly affected in PD. Focused studies of band oscillations and regional coherence enable clinicians to track disease progression and assess treatment effectiveness. EEG’s capacity to capture real-time neural activity makes it particularly effective for early detection of PD-related abnormalities [10,11].

Numerous studies have investigated machine learning and traditional methods for classifying PD using EEG data. Conventional approaches, such as statistical models, emphasize feature extraction and classification with simplified frameworks, prioritizing interpretability and computational efficiency [12,13,14]. For example, decision tree models leveraging statistical features like Hjorth parameters achieved 98% accuracy with high sensitivity and specificity [15]. Another study employed features such as mean, standard deviation, and kurtosis, allowing clinicians to infer PD presence without complex classification [16]. Budget-based models utilizing sample entropy and channel optimization reported 76% accuracy on open-eye EEG data [17]. Additionally, an ensemble method combined with an artificial neural network (ANN) and a discrete wavelet transform (DWT) yielded 98.54% accuracy [18]. Despite these achievements, handcrafted feature-based models often struggle to generalize across diverse datasets, limiting their practical utility.

Deep learning (DL) techniques have been widely explored for their capacity to discern complex patterns in EEG data. Convolutional neural networks (CNNs) and autoencoders are frequently employed to extract high-dimensional spatial and temporal features [19,20,21,22,23]. For instance, a CNN combined with long short-term memory (LSTM) layers achieved 96.9% accuracy in differentiating individuals with PD from healthy controls (HC) [24]. Capsule networks, designed to preserve spatial hierarchies in EEG data, recorded an accuracy of 89.34% [25]. Other methods include CNNs applied to Mel-spectrogram-transformed EEG data, yielding 97% accuracy [26], and approaches integrating CNNs with deep neural networks (DNNs) and ensemble empirical mode decomposition (EEMD) features, achieving 98% accuracy [27]. While DL methods offer flexibility in capturing intricate EEG patterns, they often demand significant computational resources and may yield lower accuracies, yet their ability to handle data variability makes them valuable for PD classification.

Multimodal and nonlinear analysis methods enhance PD classification by integrating EEG with other physiological and behavioral data. Techniques such as delay differential analysis [28], Graph Convolutional Networks (GCNs) [29], and forged channel CNNs [30] underscore the importance of combining EEG with complementary modalities to improve diagnostic outcomes. These approaches emphasize the need for models that achieve high accuracy, robust generalization, and resilience to data variability.

Despite progress, current methods for PD detection have limitations. Handcrafted statistical models, though accurate, often fail to generalize across diverse datasets. DL and multimodal approaches, while adaptable, can be sensitive to noisy data and inter-subject variability. Thus, an effective PD detection model must deliver high accuracy alongside robust generalization to diverse and challenging datasets.

To address these challenges, this study introduces a convolutional transformer enhanced sequential model (CTESM), integrating CNNs for spatial feature extraction, transformer blocks for temporal attention, and LSTM layers for sequential pattern retention. This architecture effectively captures contextual dependencies in EEG data, utilizing biologically informed features such as spectral power, frequency band ratios, and wavelet coefficients. The contributions of the study include

- The incorporation of transformer blocks to identify critical temporal features linked to PD-related EEG anomalies;
- The enhanced extraction of spatial and temporal features through the combined strengths of CNNs, transformers, and LSTMs;
- Improved model adaptability across diverse datasets, driven by transformer-enabled contextual analysis.

The rest of the paper is structured as follows: Section 2 details the methodology, encompassing the mathematical framework and algorithm design; Section 3 reports the classification results, evaluates the model’s strengths and limitations, and explores future research directions; and Section 4 concludes the study.

## 2. Materials and Methods

**Figure 1:**
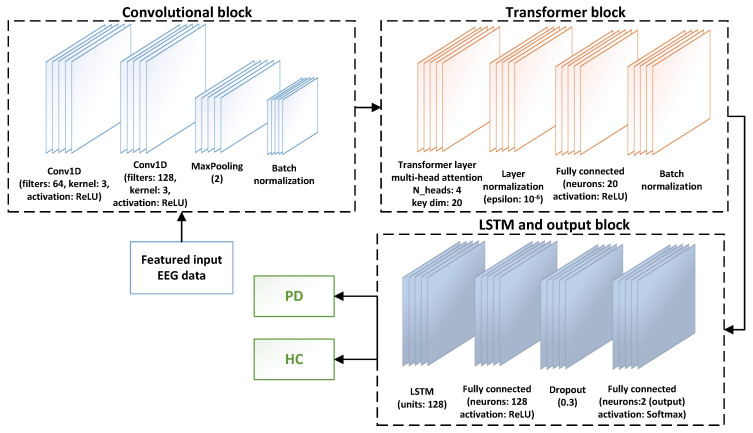
The CTESM architecture for PD detection. It integrates CNNs for spatial feature extraction, transformer blocks for temporal attention, and LSTM layers for sequential pattern analysis, facilitating precise classification of PD and HC by capturing spatial, temporal, and sequential EEG dependencies.

The architecture of the CTESM, integrating CNNs, transformer blocks, and LSTM layers, is depicted in Figure 1. The detailed procedure is described in Algorithm 1. The system design adopts a structured approach with three core stages: feature extraction, model training, and performance evaluation. Initially, spectral and temporal dependencies are extracted from raw EEG data, forming a feature matrix rich in biologically relevant patterns. These features are then used to train the CTESM, optimizing its parameters to differentiate PD from HC. Finally, the model undergoes rigorous testing, with performance assessed through accuracy, precision, recall, and F1-score metrics.

### 2.1. Dataset

This study utilizes the UC San Diego resting-state EEG dataset (Dataset 1), comprising recordings from individuals with PD and HC [31,32,33,34,35]. Table 1 summarizes its key attributes. With detailed demographic and clinical data from both groups, this dataset provides a robust foundation for evaluating the CTESM.
**Algorithm 1** Feature extraction and model training**Require:** EEG data $X_{\mathrm{EEG}}$, number of frames *N*, number of channels $N_{\mathrm{Ch}}$
**Ensure:** Model performance metrics: accuracy, precision, recall, and F1-score
  1:**Input:** Raw EEG data $X_{\mathrm{EEG}}$  2:**Initialize:** Feature matrix F←∅  3:**Windowing:** Segment $X_{\mathrm{EEG}}$ into frames $x_{{\mathrm{EEG}}_{w_{i}}}$, i=1,…,N  4:**for**  i=1 to *N* **do**  5:  **for**  j=1 to $N_{\mathrm{Ch}}$ **do**  6:         Extract the following features from each frame-channel pair $x_{{\mathrm{EEG}}_{w_{i},j}}$:              Spectral power in frequency bands              Beta-to-alpha power ratio              Median frequency              Spectral entropy              Wavelet coefficients              Approximate entropy              Statistical features: skewness, kurtosis, and zero-crossing rate  7:         Append features to F←F∪{extractedfeatures}  8:   **end for**  9:**end for**10:**Train/test split:** Divide F into training and testing sets11:**Model training:** Train model on training set, obtain predictions $\hat{y}$12:**Evaluation:** Compute performance metrics13:**Output:** Model evaluation metrics: accuracy, precision, recall, and F1-score


### 2.2. Features Extraction

To enhance accuracy and generalization, this study employs a comprehensive, biologically informed feature extraction approach. Features are meticulously selected to capture distinct EEG patterns, focusing on frequency bands, signal complexity, and nonlinear dynamics. The EEG data $x_{\mathrm{EEG}}$ are segmented into overlapping 2 s frames with a 1 s overlap, where each frame $x_{{\mathrm{EEG}}_{w_{i}}}$ (i=1,2,…,N) serves as a temporal segment for analysis. Extracted features encompass spectral power across delta, theta, alpha, beta, and gamma bands, inter-band power ratios, median frequency, spectral entropy, wavelet coefficients, and approximate entropy, supplemented by statistical measures such as skewness, kurtosis, and zero-crossing rate to characterize signal morphology and dynamics.

Spectral power: A key feature for identifying PD patterns, spectral power highlights oscillatory activity across frequency bands, notably delta, theta, alpha, beta, and gamma, with PD-related disruptions often prominent in the beta band. It is computed as [36]

(1) $$P_{\mathrm{band}}\left(x_{{\mathrm{EEG}}_{w_{i}}}\right)=\frac{1}{\left|F\right|}\sum_{f\in F}PSD\left(f\right)$$

where PSD(f) denotes the power spectral density at frequency *f* within the band range *F*, calculated for each frame $x_{{\mathrm{EEG}}_{w_{i}}}$ using the Welch method.

Band power ratio: The beta-to-alpha power ratio serves as a discriminative feature for assessing motor and cognitive disruptions. It is calculated as [37]

(2) $${\mathrm{Ratio}}_{\beta /\alpha }\left(x_{{\mathrm{EEG}}_{w_{i}}}\right)=\frac{P_{\beta }\left(x_{{\mathrm{EEG}}_{w_{i}}}\right)}{P_{\alpha }\left(x_{{\mathrm{EEG}}_{w_{i}}}\right)}$$

where $P_{\beta }$ and $P_{\alpha }$ represent spectral power in the beta and alpha bands, respectively.

Median frequency: This metric identifies dominant frequency components in EEG signals, providing insights into neurological conditions. It is defined as

(3) $$f_{\mathrm{median}}\left(x_{{\mathrm{EEG}}_{w_{i}}}\right)=f\;\mathrm{such}\;\mathrm{that}\;\sum_{k=0}^{f}PSD\left(k\right)\approx 0.5\cdot \sum_{k=0}^{f_{\max}}PSD\left(k\right)$$

where $f_{\max}$ is the maximum frequency and the cumulative sum reaches half the total power.

Spectral entropy: This quantifies frequency distribution complexity, capturing PD-related alterations. It is computed as [38]

(4) $$H\left(x_{{\mathrm{EEG}}_{w_{i}}}\right)=-\sum_{f}\left(\frac{PSD(f)}{\sum PSD}\right)\log_{2}\left(\frac{PSD(f)}{\sum PSD}\right)$$

where PSD(f) is the power at frequency *f* in $x_{{\mathrm{EEG}}_{w_{i}}}$.

Wavelet coefficients: These capture transient oscillations within specific frequency bands, emphasizing time-localized signal changes. The average wavelet coefficient $C_{j}$ at decomposition level *j* is calculated as [39,40,41]

(5) $$C_{j}\left(x_{{\mathrm{EEG}}_{w_{i}}}\right)=\mathrm{Mean}\left(|\mathrm{Wavelet}\;\mathrm{decomposition}\;\mathrm{level}\;j|\right)$$

Approximate entropy: This measures time-series regularity and predictability, with lower values indicating more predictable patterns. It is defined as [42]

(6) $$\mathrm{ApEn}\left(x_{{\mathrm{EEG}}_{w_{i}}}\right)=-\sum_{j}p_{j}\log\left(p_{j}\right)$$

where $p_{j}$ represents the probability of specific amplitude distributions in $x_{{\mathrm{EEG}}_{w_{i}}}$.

Statistical features: Features such as skewness (Skew), kurtosis (Kurt), and zero-crossing rate (ZCR) elucidate signal morphology. They are calculated as [43]

(7) $$\mathrm{Skew}\left(x_{{\mathrm{EEG}}_{w_{i}}}\right)=\frac{1}{N}\sum_{n}{\left(\frac{x_{{\mathrm{EEG}}_{w_{i}}}\left[n\right]-\mu }{\sigma }\right)}^{3}$$

(8) $$\mathrm{Kurt}\left(x_{{\mathrm{EEG}}_{w_{i}}}\right)=\frac{1}{N}\sum_{n}{\left(\frac{x_{{\mathrm{EEG}}_{w_{i}}}\left[n\right]-\mu }{\sigma }\right)}^{4}$$

(9) $$\mathrm{ZCR}\left(x_{{\mathrm{EEG}}_{w_{i}}}\right)=\frac{1}{N-1}\sum_{n=1}^{N-1}{⊯}_{\left(x_{{\mathrm{EEG}}_{w_{i}}}\left[n\right]\cdot x_{{\mathrm{EEG}}_{w_{i}}}\left[n-1\right]<0\right)}$$

where ⊯ is an indicator function for sign changes and μ and σ are the mean and standard deviation of $x_{{\mathrm{EEG}}_{w_{i}}}$, respectively.

This comprehensive feature extraction approach leverages biologically informed and statistical patterns to optimize the CTESM’s performance, facilitating precise differentiation of PD and HC.

### 2.3. Data Augmentation Strategy

To address the limited size of the participant pool, a biologically informed feature-level augmentation strategy was implemented to enhance the variability of time-series EEG representations while preserving physiological validity. Following segmentation and feature extraction from each EEG time window, synthetic instances were generated using a three-step augmentation process.

First, zero-mean Gaussian noise (σ=0.05) was added to simulate signal perturbations that naturally occur in EEG recordings. Second, temporal scaling was applied by multiplying the features with a random factor drawn uniformly from the range [0.9,1.1], mimicking amplitude fluctuations without altering the core temporal structure. Third, dynamic modulation was introduced by shifting feature values around the segment mean, thereby simulating inter-trial variability while maintaining feature centering.

For each original instance $X_{i}\in R^{C\times F}$, 50 synthetic variations were created, resulting in a dataset expansion from *N* to approximately N×51 instances. All augmented samples retained their original class labels, allowing the model to learn more generalized temporal patterns representative of both PD and HC cases. This augmentation process was applied before training and significantly improved model robustness in the context of limited real-world data.

### 2.4. The Proposed CTESM

The CTESM integrates CNNs, transformer blocks, and LSTM layers to extract spatial, temporal, and sequential patterns from EEG data, facilitating robust classification of PD and HC using biologically informed features.

The input data, structured as $X_{\mathrm{EEG}}\in R^{n_{\mathrm{channels}}\times n_{\mathrm{features}}}$, first undergo convolutional processing to derive spatial features, defined as

(10) $$X_{\mathrm{CNN}}=\phi \left(\mathrm{Maxpool}\left(\phi \left(X_{\mathrm{EEG}}*W_{c}^{\left(1\right)}+b_{c}^{\left(1\right)}\right)*W_{c}^{\left(2\right)}+b_{c}^{\left(2\right)}\right)\right)$$

where ϕ represents the rectified linear unit (ReLU) activation function and $W_{c}$ and $b_{c}$ are learnable weights and biases. Batch normalization stabilizes these feature maps, mitigating internal covariate shift.

The resulting $X_{\mathrm{CNN}}$ is processed by a transformer block with a multi-head attention mechanism, using query $Q=X_{\mathrm{CNN}}W_{q}$, key $K=X_{\mathrm{CNN}}W_{k}$, and value $V=X_{\mathrm{CNN}}W_{v}$ matrices, where $W_{q},W_{k},W_{v}$ are learnable projections. The attention is computed as [44]

(11) $$\mathrm{Attn}\left(Q,K,V\right)=\mathrm{softmax}\left(\frac{QK^{T}}{\sqrt{d_{k}}}\right)V$$

where $d_{k}$ denotes the key vector dimensionality. A residual connection and normalization follow:

(12) $$X_{\mathrm{trans}}=\frac{\mathrm{Attn}\left(Q,K,V\right)+X_{\mathrm{CNN}}-\mu }{\sigma }$$

where μ and σ are the feature mean and standard deviation.

A feed-forward network then introduces nonlinearity to $X_{\mathrm{trans}}$:

(13) $$X_{\mathrm{FF}}=\frac{f\left(X_{\mathrm{trans}}\right)+X_{\mathrm{trans}}-\mu^{\prime }}{\sigma^{\prime }}$$

where $f\left(X_{\mathrm{trans}}\right)=\psi \left(W_{f}X_{\mathrm{trans}}+b_{f}\right)$, ψ is the ReLU activation, and $\mu^{\prime }$ and $\sigma^{\prime }$ are normalization parameters.

To capture sequential dependencies, $X_{\mathrm{FF}}$ is processed by LSTM layers [45], computing the hidden state:

(14) $$h_{t}=\mathrm{LSTM}\left(X_{\mathrm{FF}},h_{t-1},c_{t-1}\right)$$

where $h_{t-1}$ and $c_{t-1}$ are the previous hidden and cell states.

The LSTM output $h_{\mathrm{LSTM}}$ feeds into a dense layer with a softmax function for classification:

(15) $$\hat{y}=\frac{\exp(W_{\mathrm{out}}h_{\mathrm{LSTM}}+b_{\mathrm{out}})}{\sum_{j}\exp\left(W_{\mathrm{out}}h_{\mathrm{LSTM}}+b_{\mathrm{out}}\right)}$$

where $W_{\mathrm{out}}$ and $b_{\mathrm{out}}$ are the output layer’s weights and biases, and $\hat{y}$ represents predicted class probabilities.

This architecture effectively integrates diverse EEG features, facilitating precise PD and HC classification. Table 2 summarizes the simulation parameters for training and evaluation.

The application of a multi-head attention mechanism focusing on the attention operations over the EEG features in the proposed model has been summarized below. After the spatial features are extracted by the CNN layers, they are reshaped and passed through the transformer block. At this stage, the attention is computed across the temporal EEG segments. The key components and operations of the attention mechanism have been summarized in Table 3. These highlight how the contextual dependencies have been modeled for the EEG sequences. The temporal and sequential patterns have been treated as complementary aspects of the EEG dynamics. The temporal patterns have been captured by the transformer block that captures the contextual dependencies across different time points. This helps the model to attend to the relevant and non-adjacent segments of the EEG activities, such as the transient beta bursts in PD. In contrast, the sequential patterns are modeled by an LSTM that captures the evolution of the signal characteristics over time. These include the gradual shifts in the oscillatory activities and phase. The transformer determined those areas to focus on at the time, and the LSTM learns how these patterns are unfolded through time. This allows the model to extract the rich temporal dynamics that can lead to improved classification.

To enhance transparency regarding the internal feature representations of the CTESM model, the specific roles of the CNN, transformer, and LSTM components are explicitly clarified. The CNN layers extract spatially localized features from the input EEG matrix, capturing both short- and long-range inter-electrode correlations and frequency-specific activations, such as beta and gamma bursts across motor and temporal regions. These features encode spatial-frequency patterns and electrode-level relationships across the scalp. The transformer block applies attention over these CNN-derived features, assigning weights to temporally segmented EEG epochs and identifying segments with contextually significant neural dynamics. The resulting features reflect temporal importance scores that highlight events such as transient desynchronization or rhythmic bursts typically observed in PD. The LSTM processes the transformer output to model sequential dependencies across time, learning how temporally weighted patterns evolve, stabilize, or fluctuate. Together, the CNN captures spatial frequency encodings, the transformer identifies temporally relevant episodes, and the LSTM learns their progression, thereby facilitating an interpretable and temporally structured classification framework for PD and HC.

## 3. Results and Discussion

This section evaluates the CTESM through a statistical analysis of raw and extracted EEG features to identify patterns distinguishing PD from HC, followed by an assessment of classification performance. Ablation experiments and benchmarking against an independent dataset validate the model’s architecture and generalization. A comparison with state-of-the-art methods highlights CTESM’s advantages, and the discussion addresses interpretability, robustness, and clinical relevance.

To address the limited dataset of 31 participants, biologically informed data augmentation was applied to the extracted features before training. Synthetic variations were generated through controlled noise addition and frequency scaling, preserving physiological relevance. This augmentation diversified the training data, facilitating the model to learn broader PD and HC patterns.

### 3.1. Statistical Analysis of Raw and Extracted Features

A comprehensive statistical analysis of raw and extracted EEG data was conducted to validate the feature extraction approach and identify discriminative patterns between PD and HC, providing a foundation for enhanced classification.

For raw EEG data, high dimensionality was managed by aggregating mean values across channels, preserving essential signal information while enabling statistical comparisons. The analysis of variance (ANOVA) and *t*-tests assessed differences between groups, with results summarized in Table 4. Channels 3 and 37 showed *p*-values below 0.05, indicating potential for distinguishing PD and HC. However, most channels lacked significant differences, highlighting the need for advanced feature extraction to capture subtle distinctions.

The analysis of extracted features focused on biologically informed patterns, including spectral power across delta, theta, alpha, beta, and gamma bands, wavelet coefficients for time–frequency dynamics and statistical measures like skewness, kurtosis, and zero-crossing rates. These features amplified neural patterns associated with PD, enhancing the model’s ability to differentiate classes compared to raw data.

**Figure 2:**
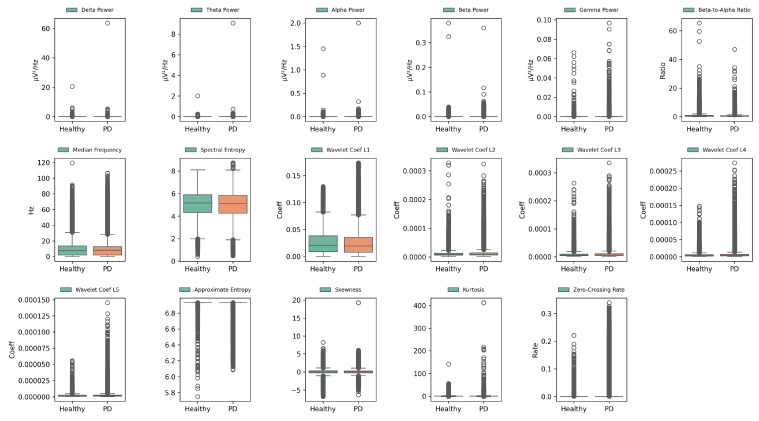
Box plots of extracted EEG features for PD and HC across all channels, illustrating variability, central tendencies, and discriminative power for enhanced class separability. Green and orange boxes represent the interquartile ranges (IQR) of the HC and PD groups, respectively. The central line indicates the median. White circles mark outliers beyond 1.5 × IQR from the quartiles.

**Figure 3:**
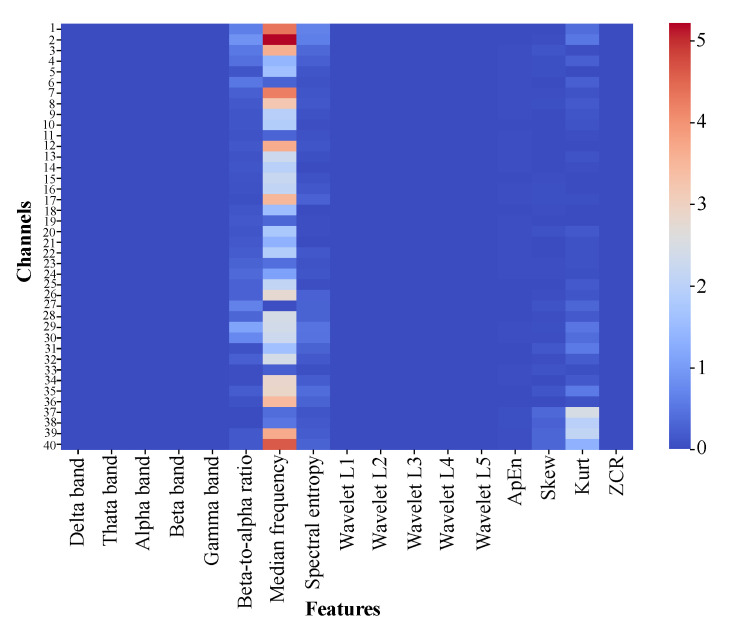
Heatmap of absolute differences in mean feature values between PD and HC across EEG channels, highlighting key regions and features for class differentiation.

Feature distributions were analyzed using box plots (Figure 2) and heatmaps (Figure 3) to elucidate their contributions in distinguishing between PD and HC. Box plots reveal variability and central tendencies of individual features across classes, highlighting significant differences. Heatmaps visualize the feature contributions across all channels and identify regions with pronounced class distinctions. Notably, the beta-to-alpha power ratios, spectral entropy, and approximate entropy were highly discriminative.

These visualizations demonstrate improved class separability through feature extraction, with clearer boundaries between PD and HC. Specific feature–channel combinations, particularly in channels 3, 15, and 37, are critical for detecting PD-related neural patterns. This underscores the value of biologically informed, frequency-specific features in capturing subtle PD patterns.

Statistical tests and visualizations collectively confirm the effectiveness of the extracted features in enhancing class discrimination. Insights from Figure 2 and Figure 3 affirm the CTESM’s potential for precise and robust PD diagnosis.

### 3.2. Model Performance Analysis

**Figure 4:**
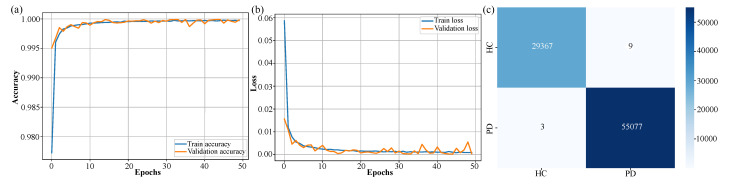
Training and validation performance of the CTESM. (**a**) Accuracy curve demonstrating steady increase to high training and validation accuracy, with no overfitting. (**b**) Loss curve indicating consistent error reduction and stable learning. (**c**) Confusion matrix highlighting exceptional test classification performance with minimal PD and HC misclassifications.

The performance of the CTESM was rigorously evaluated using training, validation, and testing metrics, focusing on accuracy and loss to assess its learning progression and generalization capabilities. As illustrated in Figure 4a, the training accuracy showed a smooth and steady increase across 50 epochs, culminating in an exceptional accuracy of 99.9%. This consistent improvement reflects the model’s ability to effectively capture intricate patterns in EEG data. Importantly, the validation accuracy closely mirrored the training accuracy throughout the epochs, demonstrating the model’s robustness and absence of overfitting.

Similarly, the loss curve presented in Figure 4b shows a consistent decline, with the loss converging to a final value of $3.8\times 10^{-4}$ by the 50th epoch. This indicates the model’s stability during training and its ability to minimize error effectively across both training and validation datasets. The evaluation on unseen test data further validated the model’s reliability and generalization capacity. Figure 4c presents the confusion matrix, highlighting exceptional classification accuracy with minimal misclassifications between the PD and HC classes. The model correctly identified 55,077 instances of PD and 29,367 instances of HC, with only minor errors in each category.

The quantitative metrics further underscore the model’s performance: an accuracy of 99.7%, precision of 99.8%, a recall of 99.7%, and an F1-score of 99.7%. These results confirm the model’s ability to generalize effectively to previously unseen data while maintaining high classification performance.

These results, depicted in Figure 4, underscore CTESM’s suitability for clinical applications. Its ability to generalize across diverse datasets, while maintaining high accuracy and precision, highlights its potential for reliable detection and classification of subtle neural patterns associated with PD. The integration of CNN, transformer, and LSTM layers in the architecture enables the model to effectively capture spatial, temporal, and sequential features in EEG data, ensuring robust and reliable performance. Stable training and validation metrics, shown in Figure 4a,b, further affirm the model’s capacity to avoid overfitting, which is an essential attribute for DL models in healthcare applications. Overall, CTESM demonstrates strong potential for deployment in real-world clinical settings.

### 3.3. Ablation Experiments and Performance Benchmarking

The CTESM was evaluated for generalization and effectiveness using a benchmarking dataset (Dataset 2) and ablation experiments. Dataset 2 [46], obtained from the University of Iowa, comprises resting-state EEG recordings from 14 individuals with PD and 14 HC. Each recording includes data from 63 EEG channels, alongside demographic and clinical attributes such as age, gender, Montreal cognitive assessment scores, unified PD rating scale motor examination scores, and disease duration for individuals with PD. This dataset provides rich multi-channel EEG data, enabling the detailed analysis of neural, cognitive, and motor function differences between PD and HC.

On Dataset 2, the CTESM achieved an exceptional accuracy of 99.9%, demonstrating its robustness in handling complex neural data and adaptability to diverse datasets beyond the original experimental setup.

**Figure 5:**
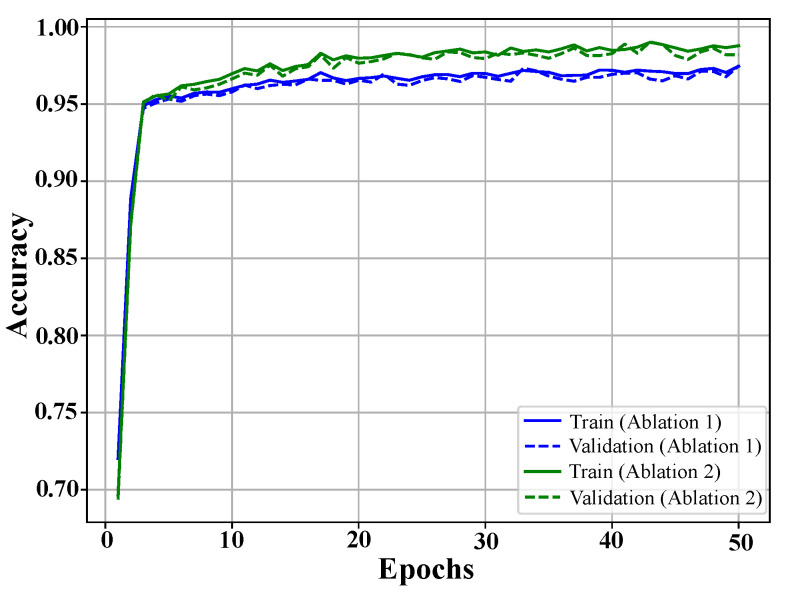
Training and validation accuracy curves for ablation studies. The blue curves represent the model without LSTM, and the green curves show the performance without regularization. Both exhibit rapid initial learning and stabilization, with regularization removal yielding higher accuracy but reduced generalization.

Ablation experiments assessed component contributions by evaluating two simplified models. Ablation 1 excluded the LSTM module, which captures sequential dependencies. Ablation 2 omitted regularization mechanisms that enhance generalization. Performance metrics are presented in Table 5, with training and validation curves shown in Figure 5.

The results showed that removing the LSTM module led to a performance drop, with an accuracy of 97.1%, a precision of 97.2%, a recall of 97.5%, and an F1-score of 96.4%. This highlights the critical role of LSTM layers in capturing long-term dependencies, which are essential for distinguishing subtle patterns in EEG signals.

Omitting regularization mechanisms resulted in slightly better performance than the LSTM removal, with an accuracy of 98.7%, a precision of 98.6%, a recall of 98.3%, and an F1-score of 98.5%. However, this configuration exhibited reduced robustness, indicating susceptibility to overfitting, especially when tested across diverse datasets.

The full model, integrating both LSTM and regularization, achieved near-perfect classification performance, reinforcing the synergistic importance of these components. The results, summarized in Table 5, validate the proposed architecture’s ability to effectively capture spatial, temporal, and sequential features in EEG data. By ensuring high accuracy and reliability across diverse datasets, the model demonstrates its potential for real-world clinical applications in PD diagnosis.

By evaluating the impact of these architectural modifications, the ablation experiments provide deeper insight into the model’s key elements, highlighting how the integration of sequential learning and robust regularization enhances performance. The results establish the CTESM as a promising tool for improving diagnostic workflows, supporting timely interventions, and enhancing patient outcomes in clinical settings.

### 3.4. Performance Comparison

The proposed CTESM was evaluated against a range of state-of-the-art models from the literature to benchmark its effectiveness in PD detection using EEG data. As summarized in Table 6, CTESM outperformed existing methods in terms of accuracy, precision, recall, and F1-score, highlighting its robustness and superior generalization capabilities.

The comparison includes models employing diverse methodologies, such as decision-tree-based models using statistical features like Hjorth parameters. These models offer high interpretability but achieve limited accuracy (98%), as they cannot capture complex temporal and spatial dependencies in EEG data. Similarly, ensemble learning methods with DWT for multi-band feature extraction attained an accuracy of 98.54%, demonstrating effectiveness in processing frequency-specific information, albeit with limitations in scalability and flexibility compared to DL approaches.

DL-based methods, such as CNN-LSTM and capsule networks, advance PD detection by learning high-dimensional spatial and temporal features directly from EEG data. For example, the CNN-LSTM model achieved an accuracy of 96.9%, and the capsule network attained 89.34%. However, these methods struggled with consistent performance across datasets and demonstrated susceptibility to overfitting.

The proposed CTESM integrates convolutional layers, transformer blocks, and LSTM modules to effectively capture spatial, temporal, and sequential dependencies in EEG data. Unlike traditional or simpler DL models, the transformer blocks in CTESM focus on critical temporal segments using an attention mechanism, enabling the detection of subtle PD-related anomalies. Additionally, biologically informed feature extraction, encompassing spectral and statistical features, enhances generalization across datasets while reducing preprocessing requirements. CTESM achieved an accuracy of 99.7%, consistently surpassing the performance of most state-of-the-art models. Its robustness under cross-validation further validates its adaptability and reliability, marking a significant step forward in EEG-based PD detection. These results, summarized in Table 6, establish CTESM as a highly reliable and scalable tool for clinical applications.

### 3.5. Discussion and Future Direction

The CTESM architecture exhibited strong capability in addressing the complexities associated with high-dimensional EEG data. By combining convolutional layers, transformer blocks, and LSTM modules, the model effectively captures spatial, temporal, and sequential information. This integration allows the transformation of subtle patterns in brain activity into meaningful and discriminative features for classifying PD.

One of the most significant strengths of the CTESM is its consistent performance across training, validation, and testing phases. The model demonstrated a reliable ability to generalize across various datasets, despite inherent variability in EEG signals that may result from differences in age, medication use, or recording conditions. This level of robustness indicates the potential of the CTESM for clinical application in real settings.

The use of biologically informed feature extraction adds further value to the model. Features such as spectral power, power ratios between frequency bands, wavelet coefficients, spectral entropy, and statistical characteristics were carefully selected to reflect neural changes commonly associated with PD. These features not only improved classification accuracy but also provided valuable insights into the underlying neural mechanisms, making them informative for clinicians who seek interpretable and evidence-based indicators for early diagnosis and disease progression monitoring.

The ability of the CTESM to detect PD-related anomalies during early stages of the condition is particularly relevant for clinical practice. Early detection plays a critical role in delaying disease progression and improving treatment outcomes. By converting complex EEG signals into actionable information, the model enables practical diagnostic support. Features such as beta-to-alpha power ratios and entropy measures also offer additional markers that can assist in visual analysis, supporting broader clinical workflows.

In addition, the CTESM supports the understanding of variability between individuals with PD and HC. This is especially important because differences in brain activity can vary significantly from one person to another. The model’s capacity to identify and interpret these differences helps not only in achieving accurate classification but also in gaining a deeper understanding of how PD affects brain function on an individual level.

In summary, the CTESM sets a new benchmark for EEG-based PD detection by integrating high accuracy, excellent generalizability, and biologically interpretable features. Its capability to manage the complexity and variability of EEG signals, while offering meaningful clinical insights, establishes it as a transformative tool for early diagnosis and personalized treatment planning. By addressing critical limitations of prior methods, the CTESM moves the field closer to accessible and effective diagnostic solutions for use in real-world healthcare environments.

While the CTESM has shown excellent classification performance, several directions remain for future exploration. The next phase of research will involve validating the model in real-time testing environments where data are collected continuously rather than in preprocessed segments. These evaluations will include testing across multiple locations and with larger and more diverse participant groups to confirm the model’s generalization capacity under various conditions.

Future work will also focus on optimizing the model for integration with portable EEG devices. This adaptation will support its use in community settings and in remote monitoring scenarios, where access to clinical infrastructure may be limited. Additionally, the incorporation of other physiological signals, such as electromyography or functional near-infrared spectroscopy, may enhance the accuracy and reliability of multimodal diagnostic frameworks.

Another priority is the development of explainability techniques that provide transparency in the model’s decision-making process. This includes the use of attention visualization, saliency mapping, and uncertainty estimation, all of which are intended to support clinical professionals in understanding and trusting the model’s predictions. These advancements will ensure that the CTESM is not only technically sound but also aligned with the expectations and requirements of healthcare practitioners.

## 4. Conclusions

The CTESM achieved a high classification accuracy of 99.7% and demonstrated strong generalization ability, surpassing previous state-of-the-art methods in distinguishing between individuals with PD and HC. By integrating biologically informed features with a sophisticated architecture that combines convolutional layers, transformer blocks, and LSTM modules, the model effectively captures spatial, temporal, and sequential patterns present in EEG data. This unified framework not only improves predictive performance but also addresses key challenges related to the complexity and variability of neural signals. The model’s consistent results during both validation and testing phases emphasize its potential as a dependable tool for clinical use. Future work will aim to validate the model in real-time environments using data from previously untrained subjects. These efforts will further assess the model’s reliability and support its translation into practical diagnostic applications for PD.

## Figures and Tables

**Table 1:** Demographic and technical specifications of Dataset 1 used for model evaluation.

Attribute	Description
Participants	31 individuals: 15 with PD (mean age 63.2 ± 8.2 years) and 16 HC (mean age: 63.5 ± 9.6 years)
Modality	Resting-state EEG
Sampling rate	500 Hz
Recording duration	5 to 10 min per session
Channels	40 electrodes (10–20 systems)
Output classes	PD and HC classification

**Table 2:** Simulation parameters of the CTESM architecture.

Parameter	Value
Epochs	50
Batch size	32
Train–test split	80% and 20%
Validation split	10%
Optimizer	Adam
Loss function	Categorical cross-entropy
Training metric	Accuracy
Testing metrics	Accuracy, precision, recall, and F1-score

**Table 3:** Summary of key operations and feature representations within the CTESM architecture.

Component	Description
CNN-extracted features	Capture spatial patterns across EEG channels, including frequency-specific activations and electrode correlations.
Transformer input	CNN feature maps reshaped into sequence format $X\in R^{T\times D}$.
Query-key-value projection	Calculated as $Q=XW_{q}$, $K=XW_{k}$, $V=XW_{v}$, with learnable parameters $W_{q},W_{k},W_{v}\in R^{D\times d_{k}}$.
Attention formula	$\mathrm{Attention}\left(Q,K,V\right)=\mathrm{softmax}\left(\frac{QK^{T}}{\sqrt{d_{k}}}\right)V$.
Multi-head attention	Parallel attention heads process inputs, with outputs concatenated and projected.
Transformer-extracted features	Capture temporal attention across EEG time windows, emphasizing contextually relevant neural activity changes.
LSTM-extracted features	Model sequential dependencies and temporal evolution, capturing rhythmic and long-term PD-related neural trends.
Model role	Facilitates the learning of long-range dependencies and frequency-specific EEG patterns.

**Table 4:** Statistical analysis results for EEG channels (*p*-values from *t*-test and ANOVA).

Channel	*t*-Test	ANOVA	Channel	*t*-Test	ANOVA	Channel	*t*-Test	ANOVA	Channel	*t*-Test	ANOVA
1	0.8786	0.8787	11	0.6720	0.6791	21	0.6407	0.6435	31	0.1257	0.0966
2	0.5376	0.4924	12	0.4453	0.3778	22	0.7896	0.7798	32	0.7076	0.7151
3	0.0310	0.0106	13	0.6611	0.6301	23	0.4836	0.4711	33	0.4185	0.4172
4	0.4213	0.3199	14	0.3137	0.2932	24	0.1887	0.2474	34	0.1493	0.1575
5	0.4280	0.3710	15	0.1010	0.1037	25	0.7716	0.7772	35	0.7444	0.7426
6	0.6031	0.5952	16	0.1372	0.1180	26	0.6583	0.6374	36	0.1755	0.1654
7	0.5447	0.5623	17	0.6015	0.5898	27	0.7412	0.7363	37	0.0602	0.0314
8	0.3361	0.2463	18	0.1611	0.1769	28	0.5017	0.4781	38	0.7718	0.7774
9	0.5205	0.5254	19	0.3729	0.4472	29	0.8472	0.8448	39	0.2659	0.2735
10	0.6203	0.6407	20	0.2521	0.1982	30	0.3772	0.3801	40	0.4388	0.4271

**Table 5:** Performance metrics for benchmarking and ablation experiments.

Metric	Dataset 2	Ablation 1	Ablation 2
Accuracy (%)	99.9	97.1	98.7
Precision (%)	99.9	97.2	98.6
Recall (%)	99.9	97.5	98.3
F1-score (%)	99.9	96.4	98.5

**Table 6:** Comparison of the CTESM with SOTA methods.

Method	Features	Accuracy (%)	Dataset
Decision tree [15]	Statistical features and Hjorth parameters	98	Dataset 1
Budget-based classification [17]	Sample entropy and channel selection optimization	76	89 PD and 89 HC (3 datasets)
ANN with DWT [18]	DWT for multi-band features	88.5	Dataset 2
CNN-LSTM [24]	Spatial and sequential EEG features	96.9	20 PD and 22 HC
Capsule network [25]	Spatial hierarchies within EEG data	89.34	55 PD and 30 HC
CNN [26]	Mel spectrogram transformed EEG	97	Dataset 1
CNN-DNN [27]	EEMD-based EEG features	98	Dataset 1
GCN [29]	Functional connectivity graphs	95.59	Dataset 2
Forged channel with CNN [30]	Smoothed pseudo Wigner-Ville distribution	90.32	Dataset 1
The proposed CTESM	Spectral, temporal, and statistical features	99.7 & 99.9	Datasets 1 and 2

## Data Availability

The code is available at https://github.com/yiamcb/CTESM (accessed on 27 April 2025).
